# A Critical Review of Carbon Quantum Dots: From Synthesis toward Applications in Electrochemical Biosensors for the Determination of a Depression-Related Neurotransmitter

**DOI:** 10.3390/ma14143987

**Published:** 2021-07-16

**Authors:** Jingying Xu, Jiangang Tao, Lili Su, Jidong Wang, Tifeng Jiao

**Affiliations:** 1 Mental Health Service Center and College of Marxism, Yanshan University, Qinhuangdao 066004, China; jingyingxu@163.com (J.X.); jiangangtao1980@163.com (J.T.); 2 Li Ren College, Yanshan University, Qinhuangdao 066004, China; lilisu@ysu.edu.cn; 3 Hebei Key Laboratory of Applied Chemistry, School of Environmental and Chemical Engineering, Yanshan University, Qinhuangdao 066004, China; 4 State Key Laboratory of Metastable Materials Science and Technology, Yanshan University, Qinhuangdao 066004, China

**Keywords:** carbon quantum dots, electrochemical biosensors, depression-related neurotransmitter, synthesis, determination

## Abstract

Depression has become the leading cause of disability worldwide and is a global health burden. Quantitative assessment of depression-related neurotransmitter concentrations in human fluids is highly desirable for diagnosis, monitoring disease, and therapeutic interventions of depression. In this review, we focused on the latest strategies of CD-based electrochemical biosensors for detecting a depression-related neurotransmitter. We began this review with an overview of the microstructure, optical properties and cytotoxicity of CDs. Next, we introduced the development of synthetic methods of CDs, including the “Top-down” route and “Bottom-up” route. Finally, we highlighted detecting an application of CD-based electrochemical sensors in a depression-related neurotransmitter. Moreover, challenges and future perspectives on the recent progress of CD-based electrochemical sensors in depression-related neurotransmitter detection were discussed.

## 1. Introduction

Neurotransmitters (NTs), as the endogenous chemical substances, are produced in different glands and transmit specific signals via chemical synapses between neurons and other cells, which help to communicate information between the brain and body [1,2]. Since the first neurotransmitter was discovered in 1921, lots of chemical messengers were found in synaptic transmission. Neurotransmitters play an important role in the mediation of behavior and cognition, such as the adjustment of muscle tone and heart rate, regulation learning, sleeping, memory, consciousness and so on. Alterations in concentration of neurotransmitters in the central nervous system are usually associated with various physical and mental changes, which would lead to many diseases, such as Huntington’s, Alzheimer’s, Parkinson’s, anxiety and depression [3,4].

Depression is a psychiatric disorder that is usually accompanied by low mood and interferes with people’s lives which can change a person’s thoughts, interests, behaviors and feelings. It is frightening that untreated depression may lead to a sustained state or suicide attempts. During diagnosis and treatment, depression-related neurotransmitters, such as the hypoactivity of monamines, especially serotonin (5-HT), noradrenaline (NA) and dopamine (DA), are found in midbrain [5]. Other neurotransmitters, for example, gamma aminobutyric acid (GABA) and glutamate, also participate in the pathogenesis of major depression [6,7,8]. Usually, the concentration of neurotransmitters is very low; for example, normal levels of DA and 5-HT in serum are less than 8.9 × 10^−13^ mol m^−3^ and 4.5 − 12 × 10^−10^ mol m^−3^, respectively [9,10]. However, the lack and imbalance of neurotransmitters would bring a significant risk of depression. Accordingly, for diagnosis, monitoring and therapeutic interventions for depression, it is highly desirable to detect NT concentrations in human fluids. To date there are many techniques using for detecting NTs, including nanopore, chemiluminescence, capillary electrophoresis, mass spectrometry, chromatography and electrophoresis [11,12,13,14,15,16,17,18]. However, laborious, expensive and complicated pretreatments are the troubles that almost all methods must be able to solve.

An electrochemical biosensor, due to its cheap, simple, highly selective, sensitive, and easy to miniaturize nature, is thought to be the most applicable detecting method for NTs in clinical treatment and diagnosis [19,20,21,22]. Especially since the appearance of novel carbon materials, significant progress has been obtained in detective research on selectivity, sensitivity and on-site detection. Among them, carbon quantum dots (or carbon dots, CDs, CQDs), due to their solubility, biocompatibility and lower toxicity, have been thought more efficient functional materials in the construction of a biosensor, since it was recognized by Sun et al., at first [23,24,25,26,27,28,29,30,31,32,33]. As semiconductor quantum dots, CDs are quasizero dimensional materials with spherical shape, and their size in three dimensions is less than 10 nm, which limited internal electrons to move in this space. The unique properties in the structure made CDs exhibit excellent features in physicochemicals and photoelectrics, which provide the possibility for the preparation of a high-quantity electrochemical sensor for use in detecting a depression-related neurotransmitter.

**Scheme 1:**
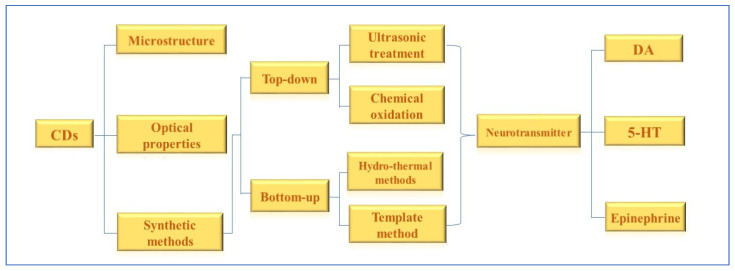
Illustration of carbon quantum dots: from synthesis toward applications in electrochemical biosensors for determination of depression-related neurotransmitters.

As shown in Scheme 1, in this review, we summarized the progress made in the past years in the field of CDs, based electrochemical biosensors for detecting depression-related neurotransmitters. We introduced the significant role of the microstructure, optical properties and cytotoxicity of CDs. The next main section covered the development of synthetic methods of CDs; that is, the “Top-down” route and “Bottom-up” route. In the concluding section, we discussed detecting the application of CD-based electrochemical sensors in depression-related neurotransmitters including dopamine, epinephrine and serotonin. Future perspectives and current challenges on the recent progress of CD-based electrochemical sensors in depression-related neurotransmitter detection were discussed as well.

## 2. Microstructure, Properties, Synthetic Methods and Biocompatibility

### 2.1. Microstructure and Properties of CDs

**Figure 1:**
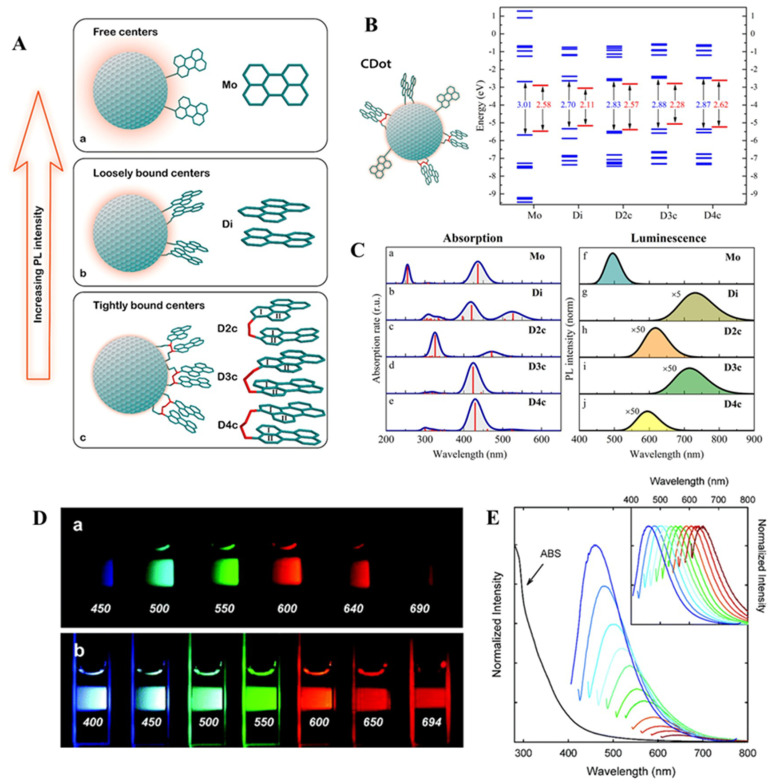
(**A**) Illustration of CDs capped with molecular-like (**a**) free, (**b**) loosely bound, and (**c**) tightly bound surface emission centers. (**B**) Energy levels of molecular orbitals of perylene-based subunits in the ground state (blue) and excited state (red). Numbers within the arrows are the energy gaps (in eV) between the HOMO and LUMO. (**C**) Absorption and PL spectra of three types of perylene−based subunits (reproduced with permission from ref. [43]) (**D**) (**a**) Filters photograph of PEG1500N− CDs was excited by 400 nm (**b**) PEG1500N− CDs was excited by different wavelengths directly (**E**) Absorption and PL spectra of PPEI−EI−CDs. Inset: emission intensities normalized to quantum yields. (Reproduced with permission from ref. [24]).

Usually, CDs were thought of as quasi-spherical nanoparticles with a distinct structure and composed of amorphous and sp^2^ carbon crystalline morphology [34,35]. Furthermore, some researchers thought that the diamond-like structure existed in CDs due to sp^3^ carbons [36]. CDs owned both core structures and complex surface groups which influenced the optical properties of CDs and water solubility [37,38]. Dopant of heteroatoms, for example, nitrogen, sulfur, changed the electronic structures and enhanced the electrical conductivity of CDs [37,39,40,41,42]. Surface modification and functionalization of CDs was performed to obtain desirable surface features and optical properties. Kundelev et al. [43] proposed a theoretical analysis on surface emission centers of CDs, which determined efficiency of photoluminescence of CDs by the verification of molecule-like subunits of polycyclic aromatic hydrocarbons (PAHs) on the CDs’ surface. They demonstrated that the PL of CDs’ decreased by about two orders of magnitude when the free monomers were replaced by the covalently bridged centers. This method provided an efficient approach to get their efficient red PL. Therefore, emissions of CDs were tunable and could cover a wide range from the visible to the near-infrared region, after the surfaces of CDs were passivated by organic or polymeric materials [24] as in Figure 1.

Optical properties of CDs were widely studied since they were accidentally discovered, because the PL of various CDs was cover from UV to visible light and even near the infrared region [44,45,46,47,48]. However, the mechanisms of tunable PL properties were not clear because inconsistent experimental observations and inaccuracy definition synthesis of different approaches made the research of luminescence more complicated. For example, the quantum confinement effect and the popular property of the semiconductor quantum dot were not always observed in CDs [49,50]. To date, intrinsic (band-gap related) and extrinsic (surface related) recombination routes were proposed to explain the luminescence mechanism of CDs [44,51,52].

The properties of CDs are largely influenced by surface groups including carboxyl and hydroxyl groups. For instance, reduction or oxidation of these groups may lead the optical properties of CDs, such as wavelength and PL intensity, to change. Rogach et al. [43] reported the synthesis of surface oxidation-related luminescence characteristics of CDs via an electrochemical route. A high surface oxidation degree of CDs generated a red-shifted emission resulting from the introduction of surface groups, which provided different emission sites on the surface of CDs. Moreover, deprotonation and protonation coming from pH adjusting, also demonstrated that surface groups were important for the photoluminescence of CDs. For example, among the research of pH-dependent behavior, the luminescence intensity decreases whenever it was at a high or low pH [53,54]. Moreover, Wen et al. [55] investigated the origin of fluorescence and carrier dynamics in CDs by an ultrafast time-resolved fluorescence technique in order to explore the fluorescence mechanism. The intrinsic and extrinsic bands, which originated from sp^2^ domains, associated with surface states to make up the fluorescence of CDs. They also demonstrated that the excitation wavelength-dependent fluorescence came from the carboxyl groups on the CDs’ surface.

In addition, for the unique band structure, controllable shape and quantum confinement effect CDs have been integrated into the construction of both the electrode and the device. Especially, for π bonds, functional moieties or dopants, CDs would be easy to react with analytes via π–π or functional group/dopants, which could facilitate charge transfer so as to increase electrochemical performance. As we know, CD-based electrochemical biosensors showed excellent properties in sensitivity due to the increased electron transfer rate. The sensitivity can be further enhanced through surface functionalization of CDs with synergistic electrochemical performances. CDs can act as substrates for polymers, enzymes and nanosheets to improve the electrochemical performance through conductivity or anchoring sites. The target can be deposited or directly grown on the surface of CDs through covalent or noncovalent interactions, which are based on the functional groups of the CDs’ surface [56,57]. Therefore, CDs have exhibited the excellent properties which would be suitable for electrochemical biosensors for use for detection of biomedicine, foods, and the environment.

### 2.2. Synthetic Methods of CDs

Usually, the synthetic method of CQD is categorized into “top-down” and “bottom-up” routes. Chemical, electrochemical, and physical methods are adopted in the “top-down” route to cleave or break down carbonaceous materials, and in the “bottom-up” route, small organic molecules [58] (or small aromatic molecules) are raw materials through the methods, such as pyrolysis, carbonization and chemical fusion, to obtain CDs [59] as shown in Table 1.

#### 2.2.1. “Top-Down” Route

In the “top-down’’ route, carbon precursors including coffee, tea, grass, graphite, carbon nanotubes and activated carbon are used to produce CDs by methods such as laser ablation, electrochemical oxidation, arc discharge, ultrasonic treatment and so on.

Xu et al. reported fluorescent CDs at first in 2004, when they proposed a preparative route to purify single-walled carbon nanotubes (SWNTs) by the eletrophoretic method. In this method, arc-discharge soot as acarbon source was oxidized by nitric acid and was extracted through agarose gel electrophoresis [60]. Zhou’s firstly prepared blue luminescent CDs by the electrochemical method result from multiwalled carbon nanotubes (MWCNTs). The electrochemical cell was composed of a carbon paper and a degassed acetonitrile solution with 0.1 M tetrabutylammonium perchlorate (TBAP) as the supporting electrolyte [61].

Ultrasonic treatment is developed to synthesize CDs. As we know, ultrasonic treatment could produce the powerful hydro-dynamic shear force through high or low pressure wave which would generate and distribute little vacuum bubbles in the liquid, cutting macroscopic carbon materials into nanoscale CDs [76,77]. Zhang et al. addressed the synthesis of the new nitrogen carbon dots (N-CDs) using a simple ultrasonic-assisted method and deposited them on the surface of BiOBr to build a photocatalyst consisting of BiOBr/N-CDs nanocomposites. N. Arsalani et al. [78] prepared the polyethylene glycol passivated fluorescent carbon dots (CDs-PEG) through a green and simple process under microwave assistance [79].

Chemical oxidation is also a popular method of CD synthesis. Carbon atoms in organic small molecules are easily inserted by acids due to their strong oxidization capability and converts them into carbonaceous materials. This process would cut organic small molecules into little sheets by controlled oxidation [80,81]. The chemical oxidation method is sensitive to reaction conditions and processes; meanwhile, it can provide CDs with excellent water solubility, fluorescence features and the possibility of large scale production. Qiao et al. [82] employed commercially activated carbon to develop a simple and effective route to prepare photoluminescent CDs facilely. The activated carbon was etched into individual CDs by treatment and passivation with nitric acid and amine-terminated compounds, such as 4,7,10-trioxa-1,13-tridecanediamine (TTDDA) or diamine-terminated oligomeric poly (ethylene glycol) H_2_NCH_2_ (CH_2_CH_2_O)_n_ CH_2_CH_2_CH_2_NH_2_ (n_av_ = 35, PEG_1500N_). The obtained CDs were high water-soluble, good biocompatibility and high quantum yield with a diameter of about 4.5 nm.

#### 2.2.2. “Bottom-Up” Route

On the other hand, the ‘‘bottom-up’’ route to synthesize CDs based on molecular precursors such as materials based on various raw materials, including protein, glucose, citric acid and chitosan, through the hydro-thermal/solvo-thermal method, the template method, the microwave assisted technique and so on [83,84,85].

Hydro-thermal/solvo-thermal methods have been considered to be simple, inexpensive, environmental-friendly methods to synthesize CDs. Usually, the Hydro-thermal/solvo-thermal system reacted by an organic precursor in a hydrothermal reactor under higher temperatures [86]. Vaibhav Naik et al. [87] reported novel nitrogen-doped CDs via the hydrothermal route. Carbon powder as a carbon source derived from acidic carbonization of sucrose combined with ammonia solution was heated in a Teflon-lined stainless-steel hydrothermal autoclave. The CDs distributed uniformly a with 10.72% QYs. Han et al. [88] demonstrated a machine-learning based technique to synthesize CDs in a hydrothermal-synthetic model which provided an insight into the successful prediction, optimization and acceleration of CDs’ synthesis process. CDs showed a high QY up to 39.3% and strong green emission.

The Template method is another popular synthesis of CDs. In this procedure, to calcine and etch the mesoporous template or silicon spheres in order to remove the support is the vital step for producing CDs. Lai et al. [68] produced CDs using mesoporous silica to use as a reactor to control the size distribution. PEG-NH_2_ and glycerol were mixed with mesoporous silica and then heated to 230 °C keeping for thirty minutes to achieve nanocomposites of CDs. Subrata Pandit et al. [89] used positional isomers of diamino benzene with citric acid under microwave to prepare CDs. Furthermore, the optical properties depended on the isomer using the soft temptations in synthesis.

### 2.3. Characterization of CDs

Due to different synthesis methods, the chemical structure and surface functional groups of each CDs will be diverse. Many methods can be exploited to characterize CQDs including transmission electron microscopy (TEM), scanning electron microscopy (SEM), X-ray diffraction (XRD), Fourier transform infrared spectroscopy (FTIR), photoluminescence, the nuclear magnetic resonance (NMR), UV spectroscopy and Raman spectroscopy.

**Figure 2:**
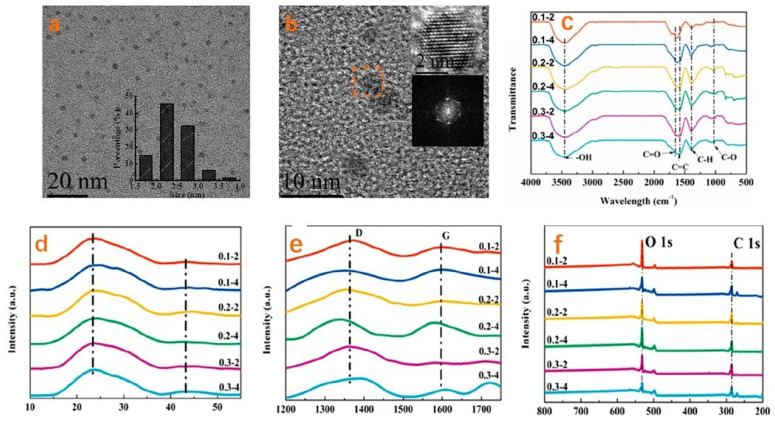
TEM (**a**), HR−TEM (**b**), FT−IR spectra of CDs (**c**), XRD pattern (**d**), Raman spectrum (**e**), XPS survey (**f**) of the CDs (sample 0.2–2), (reproduced with permission from ref. [92]).

Morphology and microstructure of CDs are investigated by TEM and SEM. TEM is usually utilized for identifying the morphology and microstructure of CDs and used to understand their shapes, sizes, dispersion because TEM possesses a high-resolution as much as 0.1–0.2 nm. XRD is also used to study particle size, phase purity and the crystal structure of CDs. When a broad peak appeared at 23°, it means a highly amorphous carbon. If two broad peaks at 25° and 44°, they are a low-graphitic carbon structure [90]. Raman spectroscopy is used to measure the lattice structure, electronic, optical and phonon properties of CDs, found near what represents the D and G bands. The peaks’ intensity ratio at 1360 and 1560 cm^−1^ under visible excitation indicated the atomic ratio between sp3 and sp2 carbon hybridization. [91] In CDs, elemental composition and bonding structure are studied by XPS and qualitative tests by FTIR and NMR. Optical properties and the electronic transition of CDs are usually determined by the measurement of fluorescence emission spectra at different excitation wavelengths and the UV−vis absorption spectrum [77]. Jing et al. used TEM, XRD, XPS and so on to investigate the microstructure and properties of CDs, which were synthesized using biomass via a hydrothermal route, as in Figure 2 [92].

### 2.4. Cytotoxicity of CDs

Assessing the cellular uptake and cytotoxicity of CDs is the first step in bio-application. It is necessary to be evaluated to gauge their biocompatibility of CDs which prepared from different carbon precursors to ensure biocompatibility. To date, amounts of research demonstrated that CDs are highly biocompatible. Hu et al. [93] established the investigation of cytotoxicity of biomass nitrogen-co-doped carbon dots (B-NCdots) prepared from the pyrolysis of natural soybean as the starting material. An MTT test was employed in human bladder carcinoma cell line (T24 cells), which indicated that the B-NCdot was low in toxicity. Sun et al. [94] developed the synthesis of CDs with the surface passivated by oligomeric PEG molecules. The toxicity of CDs in vitro was tested through the proliferation, mortality and viability of human breast cancer MCF-7 and human colorectal adenocarcinoma HT-29 cells which showed few significant toxic effects in various nanoscale configurations. In the majority of the literature, cytotoxicity data demonstrated that CDs showed very low toxicity and high intercellular transportation [95].

## 3. Detecting Application of CDs-Based Electrochemical Sensors in Depression-Related Neurotransmitter

Electrochemistry detection is a rapid, stable and low-cost technique. Usually, a biological recognition element and signal transducer system are the crucial parts in electrical measurements [96,97]. CDs have been explored to use for the electrode modifiers and carries a high signal conversion efficiency and a large surface loading rate, [98,99] using it for detecting depression-related neurotransmitter as shown in Table 2.

**Figure 3:**
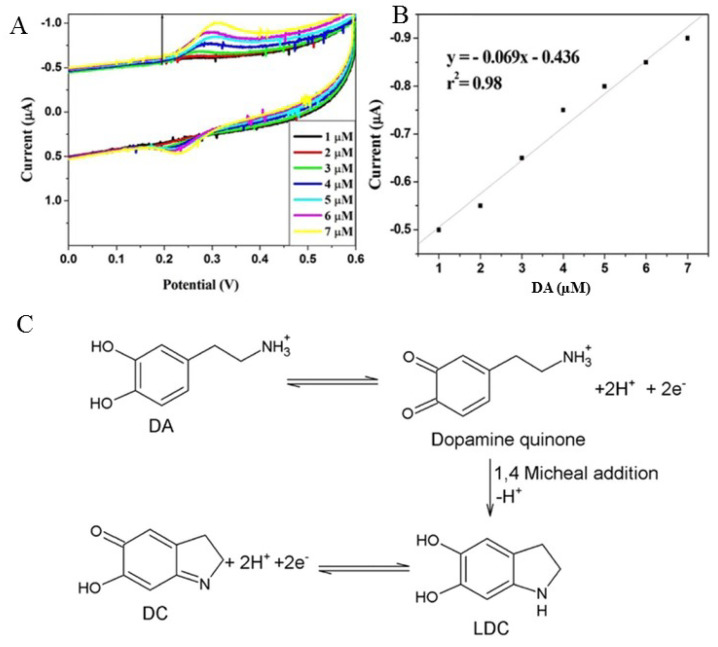
(**A**) CVs of DA with different concentration (**B**) The corresponding calibration plot for [DA] vs. Ipa. (**C**) Mechanism of electrochemical oxidation of DA on screen−printed carbon electrode/CQD film modified electrode. (Reproduced with permission from ref. [106]).

Dopamine (DA) is a kind of phenethylamine, which participated in all kinds of life process [109,110]. Algarra et al. [104] synthesized CDs via a green chemistry route to use for functionalization of a glassy carbon electrode (GCE). Based on these CDs-GCEs, the biosensor for determining dopamine was obtained with lower limits of detection (1.3 μM) and a wider linear range (0.19–11.81 μM). Compared with bare GC electrodes, the CDs-GCE showed almost 10 times better sensitivity. Notwithstanding, a higher fouling on the surface of the electrode was inevitable. Devi et al. [106] developed a route to detect dopamine by CDs’ modified electrode. CDs were synthesized by an electrochemical method which exhibited a green fluorescence. Moreover, the CDs modified screen-printed carbon electrode (SPCE) was used to construct electrochemical biosensor which showed a lower limit of detection (0.099 μM) as shown in Figure 3. Muhammad Asad et al. [105] proposed a CDs-CuO nanorod modified lead pencil electrode in which CDs came from orange peel, and detected DA with a high sensitivity. CuO nanorods provided more catalytic active sites, large surface area and short diffusion pathways, which owned highly sensitive (7 × 10^−4^ μM) and wide linear range (5–2250 μM), and was proposed to the detection of DA in deboned chicken.

**Figure 4:**
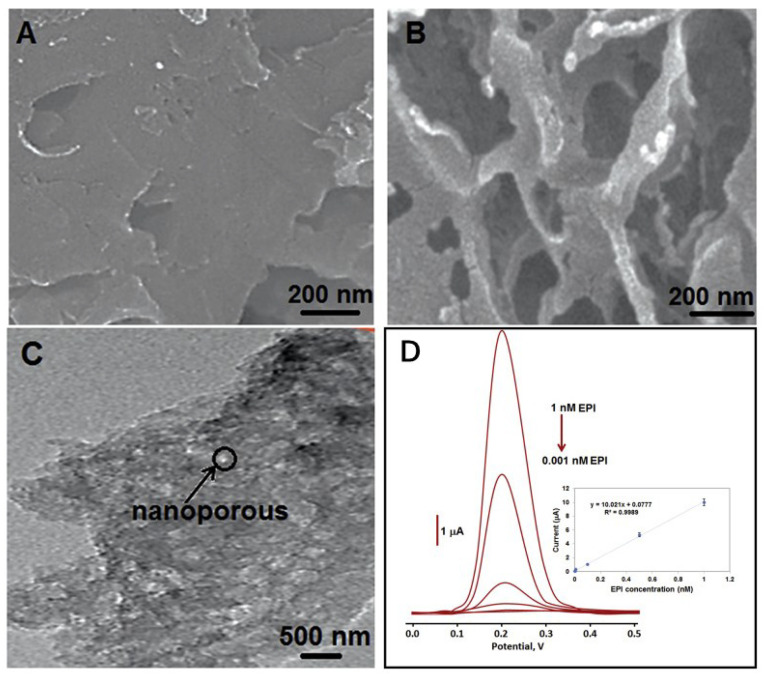
SEM image of (**A**) g-C3N4 (**B**) graphitic carbon nitride/N-doped carbon dots composite (g-C_3_N_4_/NCDS) (x) (x: 8.0% NCD_S_ (**C**) TEM image of g-C_3_N_4_/NCD_S_(x) (x: 8.0% NCDS (**D**) Detection of EPI with different concentration on the current signals with DPVs: Inset: Calibration curve of EPI concentrations at MIP/g-C3N4/NCD_S_ (8.0%)/GCE in pH 7.5 of PBS (from 1.0 × 10^−12^ to 1.0 × 10^−9^ M). (Reproduced with permission from ref. [100]).

Epinephrine (EP or Adrenaline) was secreted by the adrenal medulla to adjust the central nervous system in response to stress, anger or fear, and increased heart rate, blood pressure, cardiac output and carbohydrate metabolism. Yola et al. [100] reported an electrochemical biosensor based on graphitic carbon nitride/N-doped carbon dots composite (g-C_3_N_4_/NCDs) for epinephrine detection. The imprinted electrode was composed of g-C_3_N_4_/NCDs with 100.0 mM pyrrole and 25.0 mM epinephrine, and a cyclic voltammetry route was adopted which exhibited a lower LOD (3.0 × 10^−13^ M) and a wide linear range (1.0 × 10^−12^–1.0 × 10^−9^ M) as shown in Figure 4. Mohammad Mazloum-Ardakani et al. [101] developed an electrocatalytic biosensor for detecting epinephrine based on a carbon paste electrode resulting from derivative of hydroquinone. Differential pulse voltammetry (DPV) showed two linear dynamic ranges of 5.0 to 20.0 μM and 20.0 to 600.0 μM for EP with a lower detection limit (with 1.0 μM).

**Figure 5:**
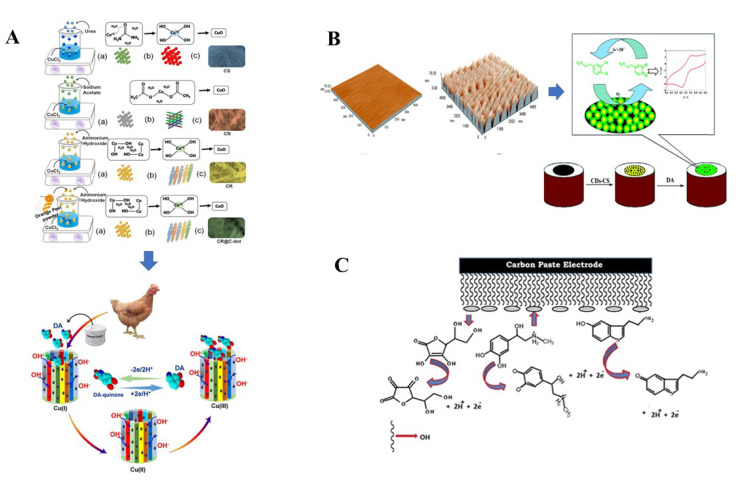
(**A**) Orange Peel Derived C-dots Decorated CuO Nanorods for the Selective Monitoring of Dopamine from Deboned Chicken (Reproduced with permission from ref. [105]. (**B**) Carbon dots and chitosan composite film based biosensor for the sensitive and selective determination of dopamine (Reproduced with permission from ref. [103] (**C**), Carbon Quantum Dot-Modified Carbon Paste Electrode-Based Sensor for Selective and Sensitive Determination of Adrenaline (Reproduced with permission from ref. [107]).

Serotonin (or 5-hydroxytryptamine, 5-HT) presented in the human brain and central nervous system which could act as a peripheral biochemical marker for depression [111]. Magdalena Kundys et al. [2] proposed an electrocatalytic oxidation route to detect neurotransmitters including dopamine, epinephrine and serotonin using a CDs modified electrodes. These CDs were deposited on the surface of electrode through a layer-by-layer approach. The electrocatalytic response was evaluated by cyclic voltammetry, differential pulse voltammetry and chronoamperometry. The electrochemical biosensor showed good electrocatalytic properties, high selectivity and high sensitivity with a lower detection limits for dopamine (0.4 mM), epinephrine (1.0 mM) and serotonin (0.8 mM) in liner range of 0.4–350 mM, 1–49 mM and 0.8–100 mM, respectively. Prasad et al. [112] introduced a simple procedure to synthesize CDs from castor oil soot using to modify the electrode. Through the voltammetry studies, they achieved a highly sensitive detection of serotonin. The improved electro-oxidation potentials was due directly to the presence of edge plane sites resulted from acid treatment of the soot. S. Sharath Shankar et al. [107] synthesized CDs from styrene and constructed biosensor to detect adrenaline (or epinephrine) and serotonin, with low detection limit of 0.004 μM as shown in Figure 5.

## 4. Conclusions and Perspectives

**Figure 6:**
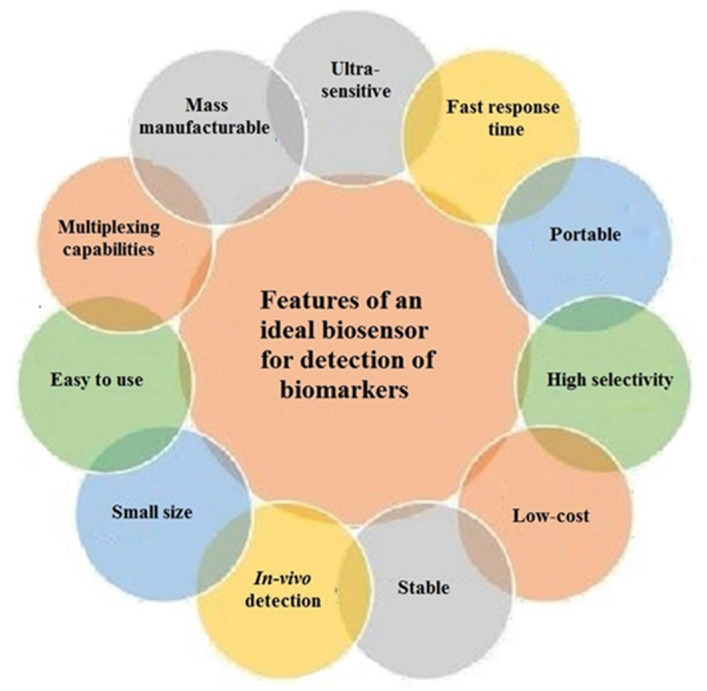
An ideal feature of an electrochemical sensor in clinics (Reproduced with permission from ref. [113]).

It played an important role to accurately detect a depression-related neurotransmitter in diagnosis, monitoring disease and therapeutic interventions of depression in clinic. Many techniques were developed to detect neurotransmitters in real samples. Electrochemical sensors were thought of as the most applicable detecting approach in clinics for its advantages including cheapness, simplicity and the fact that they are highly selective and sensitive and easy to miniaturize as shown in Figure 6 [113]. Herein, in this review, we focused on the latest strategies of CDs based electrochemical biosensors for detecting depression-related neurotransmitters. We reviewed the development of depression-related neurotransmitters, CDs and electrochemical sensors. Meanwhile, the character of CDs including their microstructure, optical properties and synthetic routes were introduced. Among them, in synthetic methods of CDs, we summarized the development of the “Top-down” route and “Bottom-up” route. Finally, we highlighted the detecting application of CD-based electrochemical sensors in a depression-related neurotransmitter including DA, EP, 5-HT and so on.

Although there are a large number of studies on CD-based electrochemical sensors in depression-related neurotransmitters, the research and application of CDs is still at the initial stage. On the synthesis of CDs, the urgent need was for high-quality. Two approaches would benefit CDs to reach large scale utilization and commercialization. One is a chemical doping way which would improve the electronic and thermal properties and electrocatalytic characteristics of CDs; the other is to combine other types of nanomaterials providing diverse properties and features.

However, it is a great surprise that CDs can be used to reduce the substantial cost of biosensors by replacing noble metal substrates in electrode materials. For the excellent properties of CDs, there is still huge space for using them as a low cost, perfect and ideal sensor material. Future research of CDs could be focused on the choice of carbon source selection and condition optimization to enhance electrochemical characteristics. Furthermore, CDs can be synthesized with other functional groups, so that on the high sensitivity of the detecting of depression-related neurotransmitters based on CD-based electrochemical sensors would be reached.

## Figures and Tables

**Table 1:** Typical synthetic methods and properties of CDs.

Route	Synthetic Method	Precursor	Diameter	QY (%)	Reference
Top-down route	Microwave synthesis	Reduced glutathione	3.8 nm	18.5	[62]
	Solvothermal	1,2,4,5-Benzenetetracarboxylic acid	5–7 nm	22	[63]
	Hydrothermal method	Dry carnation petals and polyethylenimine	2.69 ± 0.50 nm	13	[64]
	Electrochemical methods	MWCNTs	2.8 ± 0.5 nm	6.4	[61]
	Magnetic hyperthermia method	Ammonium citrate	2.4 nm	18.6	[65]
	Laser ablation	PEG1500N, PPEI-EI	5 nm	4–10	[24]
	Chemical oxidation	Activated carbon	3–4 nm	>10	[66]
Bottom-up route	One-pot, room temperature	Cetylpyridinium chloride	3.3 nm	21	[67]
	Template method	Glycerol	5.5 ± 1.1 nm	32.0	[68]
	Hydrothermal	Sulfadiazine	3.5 nm	83.2	[69]
	Reverse micelles	Glucose	0.25–0.34 nm	35.0	[70]
	Microwave-assisted pyrolysis	Date palm fruit	0.02–0.63 µm	–	[71]
	One-step ultrasonic reaction	Glucose	5 nm	7.0	[72]
	Acid oxidation	Lignin	2.4 nm	13.0	[73]
	Electrical discharge	Octane	2–5 nm	–	[74]
	Carbonization	Waste frying oil	1.0–4.0 nm	23.2	[75]

**Table 2:** A list of CDs-based sensing platforms in the detection of neurotransmitters.

Neuro-Transmitters	Electrochemical Method	Linear Range(µM)	LOD (µM)	Reference
EP	CV	1.0 × 10^−6^–1.0 × 10^−3^	3.0 × 10^−7^	[100]
	DPV	5.0–20.0	1.0	[101]
	CV	1.0–100	0.24	[102]
DA Serotonin (or 5-HT)	DPV	0.1–30	11.2	[103]
	CV	0.19–11.81	1.3	[104]
	CV	5–2250	7 × 10^−4^	[105]
	CV	1–7	0.099	[106]
	DPV	0.01–1	0.004	[107]
	CV	0.8 × 10^3^–100 × 10^3^	0.8 × 10^3^	[2]
	DPV	40–750	0.7	[108]

## Data Availability

Data is contained within the article.
